# State same‐sex marriage policies and pre‐exposure prophylaxis implementation among men who have sex with men in the United States

**DOI:** 10.1002/jia2.26180

**Published:** 2023-11-23

**Authors:** Julia Raifman, Debbie M. Cheng, Alexandra Skinner, Mark L. Hatzenbuehler, Kenneth H. Mayer, Michael D. Stein

**Affiliations:** ^1^ Department of Health Law, Policy & Management Boston University School of Public Health Boston Massachusetts USA; ^2^ Department of Biostatistics Boston University School of Public Health Boston Massachusetts USA; ^3^ Department of Epidemiology Brown University School of Public Health Providence Rhode Island USA; ^4^ Department of Psychology Harvard University Cambridge Massachusetts USA; ^5^ The Fenway Institute Boston Massachusetts USA; ^6^ Harvard Medical School Boston Massachusetts USA

**Keywords:** PrEP, men who have sex with men, structural drivers, structural interventions, stigma, policy

## Abstract

**Introduction:**

More than 70% of new HIV diagnoses in the United States were among men who have sex with men (MSM) in 2019. Pre‐exposure prophylaxis (PrEP) is a transformative innovation for reducing human immunodeficiency virus (HIV) infections. Structural stigma against sexual minorities, including in the form of state‐level policies, may affect PrEP implementation. We evaluated whether lower structural stigma reflected by earlier year of state same‐sex marriage legalization was associated with increased male PrEP prescriptions and male PrEP‐to‐need ratio (PnR), a ratio of PrEP prescriptions to new HIV diagnoses.

**Methods:**

We used 2012−2019 AIDSVu data on male PrEP prescriptions and male PnR in each US state and year. We used generalized estimating equations to evaluate the relationship between the timing of implementing state same‐sex marriage policies and the outcomes of male PrEP prescriptions per 100,000 people and the male PnR. We adjusted for calendar year, Medicaid expansion and the political party of the governor in each state.

**Results:**

State implementation of same‐sex marriage policies in earlier, relative to later, periods was associated with increases in the rate of male PrEP prescriptions and in the male PnR. Specifically, implementing state same‐sex marriage policies between 2004 and 2011 and between 2012 and 2013 were each associated with greater rates of male PrEP prescriptions relative to implementing same‐sex marriage policies between 2014 and 2015. Implementing state same‐sex marriage policies between 2004 and 2011 as well as between 2012 and 2013 were both significantly associated with a greater male PnR relative to implementing same‐sex marriage policies between 2014 and 2015. By 2019, the difference in male PrEP prescriptions was 137.9 (97.3−175.5) per 100,000 in states that implemented same‐sex marriage in 2004−2011 and 27.2 (23.3−30.5) per 100,000 in states that implemented same‐sex marriage from 2012 to 2013, relative to states that implemented same‐sex marriage in 2014−2015.

**Conclusions:**

Earlier implementation of state same‐sex marriage policies was associated with greater rates of male PrEP prescriptions. Reducing state‐level structural stigma may improve HIV prevention among MSM in the United States.

## 1 INTRODUCTION

There are an estimated 36,000 new cases of human immunodeficiency virus (HIV) each year in the United States despite preventive tools. More than 70% of new HIV acquisitions occur among men who have sex with men (MSM) [1], and the lifetime risk of HIV for MSM is estimated to be more than 80 times that of heterosexual men [2]. Understanding how structural stigma and discrimination affect PrEP implementation can inform future efforts to address HIV and other diseases.

With a transformative finding in 2010—that pre‐exposure prophylaxis (PrEP) was more than 90% effective for preventing HIV among those who adhered to it [3]—there is potential to evaluate how discriminatory policies and structural stigma affected the implementation of this HIV prevention innovation. The introduction of PrEP overlapped with another transformative change for MSM: same‐sex marriage was legal in just seven states the year PrEP was approved. By 2015, same‐sex marriage was legal in all 50 US states. Understanding how same‐sex marriage and other human rights affect PrEP implementation and HIV prevention is important for informing policies around the world. The Human Rights Campaign indicates same‐sex marriage is legal in just 34 of 195 countries [4].

Unequal rights for sexual minorities are a form of discrimination and structural stigma, defined as “the societal‐level conditions, cultural norms, and institutional policies and practices that constrain opportunities, resources and wellbeing” [5]. Fundamental cause theory stipulates that structural stigma at the broadest macro level, such as stigmatizing national and state policies, leads to stigma downstream at the level of local institutions, peers and families [6, 7]. There is evidence that national policies shape individual attitudes towards sexual minorities [8, 9], and that stigma reflected in state policies [10, 11, 12, 13], institutional policies and programmes [14], and family and peer attitudes [15, 16] shape sexual minority health, including HIV‐related outcomes [17].

Structural stigma may affect PrEP awareness and use through several mechanisms. Sexual minority stigma could drive the extent to which states and cities invested in campaigns and programmes to promote PrEP among MSM and among healthcare providers. State‐level policies could also shape healthcare provider attitudes and behaviours, such as comfort asking patients about sexual orientation [18, 19] and treatment of sexual minority patients [20, 21, 22]. Prior evidence indicates that stigma against MSM is a real concern; 65% of sexual minority healthcare providers recruited through an online survey reported hearing disparaging remarks about sexual minorities in the workplace [23]. In another study, medical student stigma against sexual minorities was associated with greater anticipation of MSM PrEP patient risk compensation and non‐adherence, beliefs which were associated with lower PrEP prescribing intentions [24].

We evaluated whether there was an association between the timing of state same‐sex marriage policies and male PrEP prescriptions in the United States. We focused on same‐sex marriage policies as a particularly salient change in sexual minority rights, with earlier adoption potentially reflecting and perpetuating lower state‐level structural stigma [5, 25]. Our study builds on prior research that supports links between sexual minority rights and health [10, 11, 13], positing a relationship between structural stigma and PrEP implementation. An analysis of cross‐sectional Internet survey data indicated living in states with lower levels of state‐level structural stigma against sexual minorities, measured as a composite score, was associated with greater PrEP awareness and use among MSM in 2013 [26]. Another analyses of these cross‐sectional data indicated that there was more PrEP awareness and use among MSM living in states that had implemented same‐sex marriage by the time of the survey [27]. In this analysis, we expand on this previous work by using longitudinal, nationwide male PrEP prescription data to evaluate the association between the timing of state same‐sex marriage policies and PrEP implementation in the United States.

## 2 METHODS

### 2.1 Objective

The objective of the analysis was to estimate the association between the period of state same‐sex marriage policies and male PrEP prescription rates in the United States.

### 2.2 Hypothesis

We hypothesized that states that implemented same‐sex marriage in earlier periods would scale up male PrEP prescriptions faster than states that implemented same‐sex marriage policies in later periods.

### 2.3 Data

We used de‐identified AIDSVu data on the number of PrEP prescriptions per 100,000 males aged 13 or over in each state, from 2012 to 2019. AIDSVu PrEP data are the most comprehensive data available on PrEP prescriptions from a commercially available sample that includes more than 54,000 pharmacies, 1500 hospitals, 800 outpatient facilities, 80,000 physician practices and some clinics in academic settings. The dataset excludes entities that do not share their data, such as closed healthcare systems like Kaiser Permanente. The publicly available dataset was produced by excluding tenofovir disoproxil fumarate/emtricitabine prescriptions for HIV treatment, post‐exposure prophylaxis and hepatitis B based on medical claims. The dataset includes state‐specific upweighting of PrEP prescriptions to account for underestimates due to misclassified prescriptions. The dataset does not contain information on the race, ethnicity, sexual orientation or gender identity of people who receive prescriptions.

We also used the AIDSVu dataset to estimate the male PrEP‐to‐need ratio (PnR), defined as the number of male PrEP users divided by new HIV diagnoses among males 13 and older in each state and year by sex [28, 29]. We used the 2019 AIDSVu dataset on new male HIV diagnoses among people 13 and older to plot the rate of male PrEP prescriptions relative to new male HIV diagnoses.

We linked AIDSVu datasets on male PrEP prescriptions and male PnR to the American Community Survey 1‐year population estimates of males aged 13 and older, using the estimates pertinent to each calendar year. To derive male sexual minority population estimates, we multiplied estimates of the male population aged 13 and older by the University of California, Los Angeles Williams Institute data on the adult lesbian, gay, bisexual and transgender population in each US state [30].

We limited the samples for all analyses to people who indicated male sex. While PrEP is indicated for any males who have sex partners of unknown HIV status and do not always use condoms or who are people who inject drugs (PWID) [31], prior analyses indicate that male PrEP users are predominantly MSM. An analysis of PrEP users in a California hospital system indicated that 99% were MSM [32]. Other analyses indicate that PrEP use is low among PWID [33, 34], even though PrEP is a promising approach to preventing HIV for PWID.

### 2.4 Exposure

The main exposure of interest was the year of state same‐sex marriage policy implementation, ranging from 2004 in Massachusetts to 2015, when the US Supreme Court made same‐sex marriage legal in all 50 states. We considered states to have implemented same‐sex marriage laws if same‐sex marriage was legal by the first of January in each year. We grouped states into those that implemented same‐sex marriage between 2004 and 2011, in 2012–2013, and in 2014–2015 to ensure there were at least five states in each group. We used binary indicators for each of the two earlier time periods (before 2012 and 2012–2013) relative to the latter time period (2014 and 2015).

### 2.5 Outcome

The main outcome of interest was the rate of male PrEP use per 100,000 males 13 or older in each state and year. A secondary outcome of interest was the male PnR.

### 2.6 Covariates

States that implemented same‐sex marriage in earlier periods may systematically differ politically and with respect to other policies from states that implemented same‐sex marriage in later periods. To account for political differences that may also affect commitment to PrEP implementation, we adjusted for a binary indicator for having a Democratic governor, relative to a Republican or Independent governor, based on National Council of State Legislators data. To account for health policies that could affect PrEP prescription rates, we adjusted for state Medicaid expansion as a binary indicator based on Kaiser Family Foundation data [35]. We included these covariates because they may be associated with both the timing of state same‐sex marriage policies and with male PrEP prescription rates and male PnR. We also adjusted for calendar year as a linear variable, given that PrEP prescriptions increased over time.

### 2.7 Analyses

We first described the timing of same‐sex marriage policy by state. We estimated and plotted population‐weighted means of male PrEP prescriptions per 100,000 people and male PnRs in each calendar year, by period of state same‐sex marriage implementation.

To evaluate the relationship between period of state same‐sex marriage policy implementation and male PrEP prescriptions, we used generalized estimating equations (GEE) negative binomial regression models to account for repeated observations of states with a log link, and log population offset. To account for the size of the sexual minority population in each state, we conducted a secondary analysis of the relationship between the period of state same‐sex marriage policy implementation and male PrEP prescriptions through GEE negative binomial regression with a log link and with a modification in which the log population offset was an estimate of the male sexual minority population. To evaluate the relationship between period of state same‐sex marriage policy implementation and male PnR, we used GEE linear regression models to account for repeated observations of states. In both analyses, we adjusted for calendar year, Medicaid expansion and Democratic governors. We weighted regressions by the population of each state. We used Stata 15.0 to conduct all analyses.

To further describe the male PnR in 2019, we plotted the male PrEP prescription rate by new male HIV diagnoses for each state in 2019. We also conducted linear regression analyses of the relationship between new 2019 HIV diagnoses and the male PrEP prescription rate separately for each period in which same‐sex marriage was implemented, adjusting for Medicaid expansion and whether the governor was a Democrat.

In a sensitivity analysis, we repeated the main analysis with 2015 as a separate period to investigate whether there were differences between states that implemented same‐sex marriage in 2014 and 2015, given that states that did not implement same‐sex marriage until the nationwide Supreme Court decision may have differed from those that implemented same‐sex marriage prior to the Supreme Court ruling.

This analysis was considered non‐human subjects research by the Boston University Institutional Review Board. Because only de‐identified data were used and the research was considered non‐human subjects research, there was no consent procedure.

## 3 RESULTS

**Figure 1:**
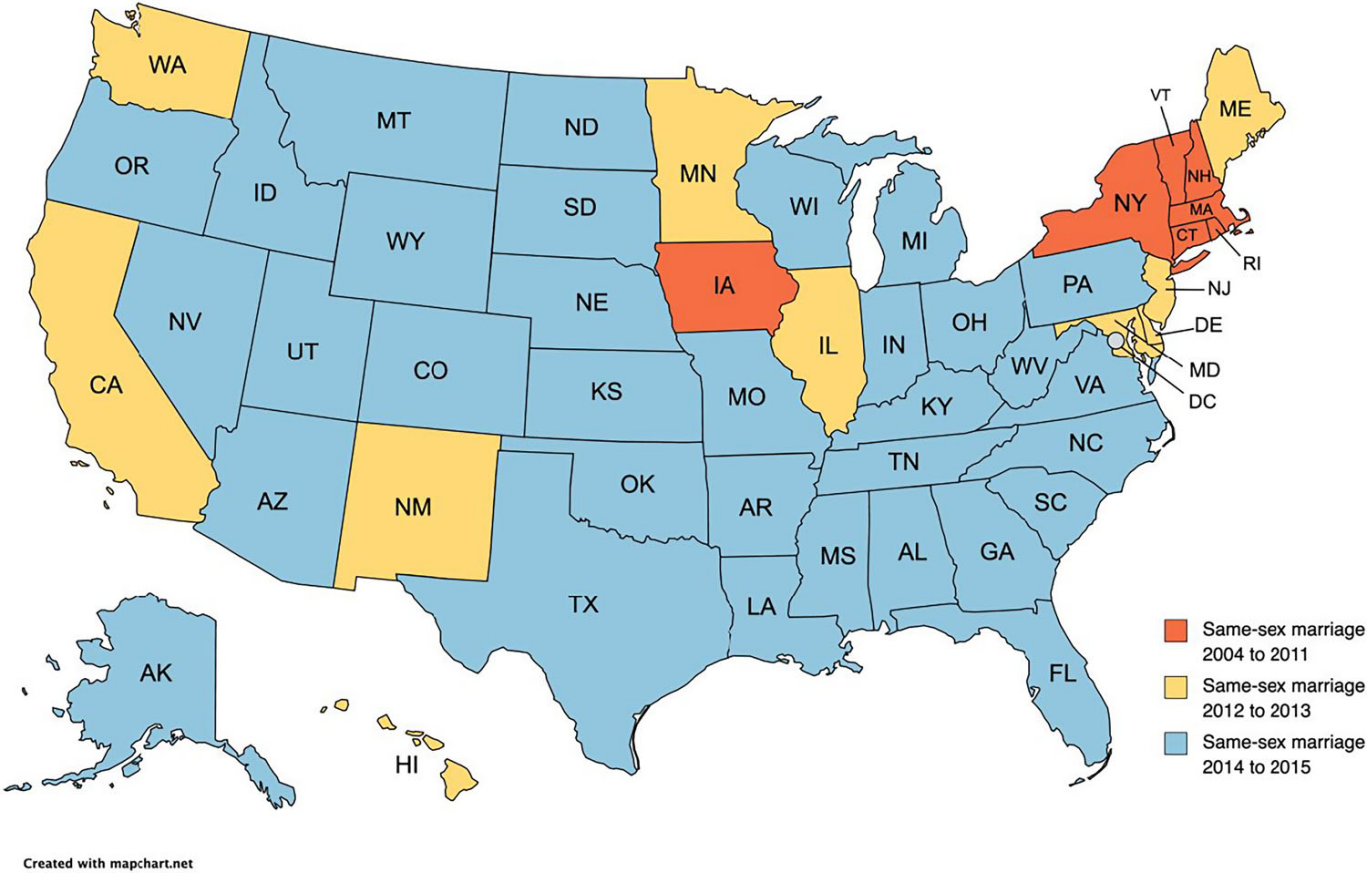
Years in which states implemented same‐sex marriage.

We observed each of 50 US states over 8 years from 2012 to 2019, summing to a total of 400 state‐years in the analysis. There were seven states that implemented same‐sex marriage before 2012, 10 states that implemented same‐sex marriage in 2012 and 2013, and 33 states that implemented same‐sex marriage in 2014 and 2015 (Figure 1). The first states to implement same‐sex marriage were more likely to be in the Northeast, whereas states that implemented same‐sex marriage in the 2012–2013 period or the 2014–2015 period were in each region of the United States.

**Figure 2:**
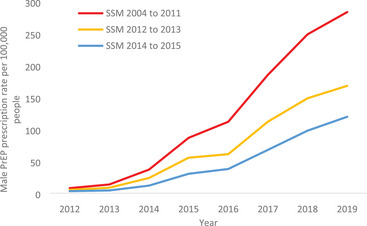
Year of state same‐sex marriage policy and male PrEP prescription rate per 100,000 people, over time. SSM, same‐sex marriage. The lines depict the total male pre‐exposure prophylaxis prescription rate for all states implementing same‐sex marriage in a given year.

There were increases in the male PrEP prescription rate in all 50 states between 2012 and 2019. The rate of male PrEP prescriptions per 100,000 people increased from a mean of 4 in 2012 to a mean of 151 in 2019 (Figure 2). By 2019, the rate of male PrEP prescriptions ranged from 36 per 100,000 in Wyoming to 352 per 100,000 in New York. PrEP prescription rates were larger in states that implemented same‐sex marriage between 2004 and 2011 and 2012–2013, relative to 2014–2015. By 2019, the male PrEP prescription rate was 238.8 in states implementing same‐sex marriage between 2004 and 2011 and 168.3 in states implementing same‐sex marriage in 2012–2013, relative to 119.8 in states implementing same‐sex marriage in 2014–2015.

**Figure 3:**
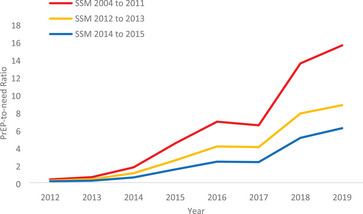
Year of state same‐sex marriage policy and male PrEP‐to‐need ratio, over time. SSM, same‐sex marriage. The lines depict the total male pre‐exposure prophylaxis‐to‐need‐ratio for all states implementing same‐sex marriage in a given year.

The male PnR increased from less than 1.0 in all states in 2012 to a mean of 9.0 in 2019 (Figure 3). The range in 2019 was from less than 3 in Mississippi and South Carolina to more than 20 in New Hampshire. The male PnR was larger in states that implemented same‐sex marriage between 2004 and 2011 and 2012–2013, relative to 2014–2015. By 2019, the total male PnR was 15.6 in states implementing same‐sex marriage between 2004 and 2011 and 8.8 in states implementing same‐sex marriage in 2012–2013, relative to 6.2 in states implementing same‐sex marriage in 2014–2015.

Based on adjusted regression analyses, implementing state same‐sex marriage policies between 2004 and 2011 (rate ratio: 2.27, 95% CI: 1.71−3.02) and between 2012 and 2013 (rate ratio: 1.40, 95% CI: 1.12−1.76) were each associated with greater rates of male PrEP prescriptions relative to implementing same‐sex marriage policies between 2014 and 2015 (Table 1). Estimates of the relationship between period of same‐sex marriage implementation and male PrEP prescription rate were consistent with the main results but of a lesser magnitude in secondary analyses where estimates of the male sexual minority population were used as offsets in the model. In this analysis, implementing state same‐sex marriage policies between 2004 and 2011 (rate ratio: 1.85, 95% CI: 1.45−2.35) and between 2012 and 2013 (rate ratio: 1.21, 95% CI: 1.01−1.45) were each associated with greater rates of male PrEP prescriptions relative to implementing same‐sex marriage policies between 2014 and 2015 (Table 1). In linear analyses of PnR, implementing state same‐sex marriage policies between 2004 and 2011 (PnR: 1.26, 95% CI: 0.15−2.36) and between 2012 and 2013 (PnR: 3.83, 95% CI: 2.84−4.81) were each associated with a greater male PnR relative to implementing same‐sex marriage policies between 2014 and 2015.

**Table 1:** Regression estimates of the relationship between period of same‐sex marriage, male PrEP prescription rates and male PrEP‐to‐need ratios

	Male PrEP prescription rate per 100,000 males		Male PrEP prescription rate per 100,000 gay or bisexual males		Male PrEP‐to‐need ratio	
	Adjusted incidence rate ratio	95% confidence interval	Adjusted incidence rate ratio	95% confidence interval	Adjusted mean difference	95% confidence interval
Year of same‐sex marriage						
2004–2011	2.27	1.71–3.02	1.85	1.45–2.35	1.26	0.15–2.36
2012–2013	1.40	1.12–1.76	1.21	1.01–1.45	3.83	2.84–4.81
2014–2015	Reference		Reference		Reference	
Year	1.65	1.59–1.70	1.65	1.60–1.71	1.09	0.85–1.33
Medicaid expansion	1.56	1.32–1.83	1.50	1.28–1.76	0.43	−0.53 to 1.38
Democratic governor	0.91	0.80–1.04	0.90	0.79–1.03	−0.24	−1.25 to 0.78

*Note*: For male pre‐exposure prophylaxis prescription rates per 100,000, generalized estimating equations negative binomial regression models were used to account for repeated observations of states with a log link, and log population offset. For male pre‐exposure prophylaxis‐to‐need‐ratio, we used generalized estimating equations linear models to account for repeated observations of states.

**Figure 4:**
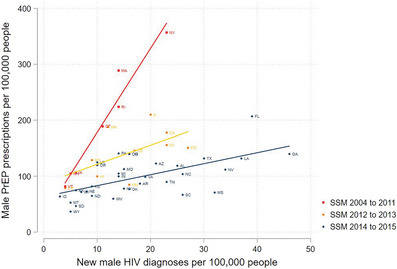
New HIV diagnoses among males and male PrEP prescription rate for persons 13 years or older, by year of same‐sex marriage policy implementation. SSM, same‐sex marriage. There was a positive correlation between new human immunodeficiency virus diagnoses and male pre‐exposure prophylaxis prescriptions per 100,000 people overall, with a stronger correlation in states that implemented same‐sex marriage policies earliest.

By 2019, there was a positive correlation between new HIV diagnoses per 100,000 males and PrEP prescriptions per 100,000 males (Figure 4). The increase in male PrEP prescriptions per increase in HIV diagnoses was larger in states that implemented same‐sex marriage policies between 2004 and 2011 (17.2, 95% CI: 12.2−22.2) than in those that implemented same‐sex marriage policies in 2014–2015 (2.0, 95% CI: 0.0−2.9). There was not a significant association between new HIV diagnoses per 100,000 males and PrEP prescriptions per 100,000 males in states that implemented same‐sex marriage in 2012 and 2013 (3.6, 95% CI: −3.3 to 10.5).

In the sensitivity analysis repeating the main analysis with 2015 as a separate period, we did not detect a difference between male PrEP implementation in 2014 relative to 2015 (rate ratio: 1.10, 95% CI: 0.77−1.57), while implementing state same‐sex marriage policies between 2004 and 2011 (rate ratio: 2.39, 95% CI: 1.74−3.27) and between 2012 and 2013 (rate ratio: 1.47, 95% CI: 1.12−1.93) remained associated with greater rates of male PrEP prescriptions relative to implementing same‐sex marriage policies in 2015 (Table S1). In the secondary sensitivity analysis using an estimate of the male sexual minority population as the offset, implementing same‐sex marriage in 2004–2011 was significantly associated with the male PrEP prescription rate (rate ratio: 1.84, 95% CI: 1.40−2.42), while implementing same‐sex marriage in 2012–2013 was not (rate ratio: 1.21, 95% CI: 0.97−1.52). In the PnR analysis, implementing state same‐sex marriage policies between 2004 and 2011 (PnR: 58.56, 95% CI: 21.09−162.63) and between 2012 and 2013 (4.48, 95% CI: 1.43−13.98) were each associated with a greater male PnR relative to implementing same‐sex marriage policies between 2014 and 2015.

## 4 DISCUSSION

We studied the association between two transformative developments for sexual minority men in the United States—equal marriage rights and the introduction of PrEP as a novel, highly effective approach to HIV prevention. Data collected since the start of PrEP availability in 2012 make it possible to evaluate how different policy environments have shaped the full course of PrEP implementation. We studied the period of state same‐sex marriage policies as an indicator of reduced structural stigma. We found that state implementation of same‐sex marriage policies in earlier, relative to later, periods was associated with increases in the rate of male PrEP prescriptions and in the male PnR.

Throughout the study period and across specifications, US states that implemented same‐sex marriage policies in 2004–2011 had higher rates of male PrEP prescriptions and male PnR than states that implemented same‐sex marriage policies in 2014 or as a result of the federal Supreme Court decision in 2015. As PrEP prescription rates and male PnR increased across all states from 2012 to 2019, the absolute difference grew between states implementing same‐sex marriage in different periods. It is possible that structural stigma at the time of PrEP introduction played a formative role in early investments and efforts towards PrEP implementation. It is also possible that earlier implementation of same‐sex marriage policies is a reflection of enduring state‐level policy climates surrounding sexual minorities. State‐level structural stigma shapes institutional [36], interpersonal [37] and internalized [38] stigma, each of which could affect MSM patient care and PrEP prescriptions. At the same time, implementing same‐sex marriage policies was not enough to reduce the gap in PrEP prescriptions across states that implemented same‐sex marriage policies in later periods. There is a need for further work to scale up PrEP implementation in these states.

The finding that state same‐sex marriage policies were associated with PrEP prescriptions is consistent with prior evidence indicating that state‐level policies related to sexual orientation are associated with changes in sexual minority health disparities [10, 11, 12, 13]. The findings also add longitudinal, nationwide evidence to a body of literature indicating that state‐level structural stigma towards sexual minorities affects HIV prevention [25, 26, 27, 39]. We also found that Medicaid expansion was associated with increased PrEP, underlining the importance of other policies that counter structural inequities for MSM.

In 2019, there was a positive correlation between new HIV diagnoses and PrEP prescriptions, suggesting that PrEP was reaching states with the most need for it; however, this correlation was much stronger in states that implemented same‐sex marriage earlier relative to later. The states that implemented same‐sex marriage in later periods are also the states with the most new HIV diagnoses. There remains a need for further investment in PrEP implementation and structural supports such as Medicaid expansion in states with the highest new HIV diagnoses.

This study has both strengths and limitations. A strength of the study is that it is based on a nationwide dataset of PrEP prescriptions, while a limitation is that the dataset omits some closed health systems, potentially restricting generalizability. Our findings are descriptive rather than causal and are vulnerable to confounding by unmeasured differences between states. Statistical power and inclusion of covariates were limited by the number of US states (50). Although the AIDSVu datasets have information on patient sex, they lack information on patient race/ethnicity as well as sexual orientation and gender identity, and some patients and PrEP users with male sex may identify as transgender women. We also lacked precise information on the population denominator of MSM, and this could vary by state. While we used estimates of the gay and bisexual population, these estimates were not disaggregated by sex at birth and do not capture all MSM, given that some MSM may identify as straight. The majority of male PrEP users are MSM [31], suggesting that our analysis is mostly capturing this population. However, given that structural racism and racial discrimination shape stark racial disparities in HIV and in PrEP use among MSM [2], future research is needed to determine whether the relationships observed are similar across racial/ethnic subgroups of MSM. Potential future analyses could explore the relationship between sexual minority rights and PrEP use by race and ethnicity in datasets that contained this demographic information, or analyses of state same‐sex marriage and HIV incidence by race and ethnicity. Analyses of how structural stigma and structural racism affect intersectional populations are also important [40]. An additional limitation is that we lack individual‐level identifiers and cannot account for within‐person correlation for individuals with PrEP prescriptions in multiple years.

## 5 CONCLUSIONS

Earlier implementation of state same‐sex marriage policies between 2004 and 2011 and between 2012 and 2013 was associated with greater rates of male PrEP prescriptions relative to later implementation of same‐sex marriage in 2014 and 2015. Implementation of state same‐sex marriage policies between 2004 and 2011 was also associated with greater male PnR relative to later implementation of same‐sex marriage in 2014 and 2015. The findings are aligned with fundamental cause theory [6, 7] and prior literature on the relationship between structural stigma and HIV among MSM [26, 27, 41]. Policies and practices that reduce structural stigma against MSM may reduce health disparities among sexual minority relative to heterosexual populations.

## AUTHORS’ CONTRIBUTIONS

JR designed the study, obtained the data, conducted the statistical analysis and drafted the manuscript. DMC, AS, MLH, KHM and MDS contributed to the conceptualization of the study, interpreting data and critical revision of the manuscript.

## FUNDING

This study was funded by grants from the National Institute of Mental Health (K01 MH116817) and the Providence/Boston Center for AIDS Research (P30AI042853).

## COMPETING INTERESTS

The authors have no competing interests to declare.

## Supporting information

Table S1: Regression estimates of the relationship between period of same‐sex marriage implementation, male PrEP prescription rates and male PrEP‐to‐need ratios

## Data Availability

The AIDSVu data used as the main outcome for this study are publicly available at aidsvu.org.
