# Supplementary material for: State same‐sex marriage policies and pre‐exposure prophylaxis implementation among men who have sex with men in the United States
Source: J Int AIDS Soc. 2023 Nov 23;26(11):e26180. doi: 10.1002/jia2.26180 (PMC10667585; doi:10.1002/jia2.26180)
Supplement: Supplementary file 1 — Table S1: Regression estimates of the relationship between period of same‐sex marriage implementation, male PrEP prescription rates and male PrEP‐to‐need ratios [file JIA2-26-e26180-s001.docx]

**Appendix Table 1: Regression estimates of the relationship between period of same-sex marriage implementation, male PrEP prescription rates, and male PrEP-to-need ratios**

|  | **Male PrEP Prescription Rate per 100,000 males** | | **Male PrEP Prescription Rate per 100,000 gay or bisexual males** | | **Male PrEP-to-Need Ratio** | |
| --- | --- | --- | --- | --- | --- | --- |
|  | Adjusted incidence rate ratio | 95% confidence interval | Adjusted incidence rate ratio | 95% confidence interval | Adjusted mean difference | 95% confidence interval |
| **Year of same-sex marriage** |  |  |  |  |  |  |
| **2004 to 2011** | 2.39 | 1.74 - 3.27 | 1.84 | 1.40 - 2.42 | 58.56 | 21.09 - 162.63 |
| **2012 to 2013** | 1.47 | 1.12 - 1.93 | 1.21 | 0.97 - 1.52 | 4.48 | 1.43 - 13.98 |
| **2014** | 1.10 | 0.77 - 1.57 | 1.00 | 0.73 - 1.35 | 1.56 | 0.77 - 3.17 |
| **2015** | Reference |  | Reference |  | Reference |  |
| **Year** | 1.65 | 1.59 - 1.70 | 1.65 | 1.60 - 1.71 | 2.98 | 2.34 - 3.79 |
| **Medicaid expansion** | 1.55 | 1.32 - 1.83 | 1.50 | 1.28 - 1.76 | 1.52 | 0.59 - 3.91 |
| **Democratic governor** | 0.91 | 0.79 - 1.04 | 0.90 | 0.78 - 1.04 | 0.77 | 0.28 - 2.12 |

Notes: For male pre-exposure prophylaxis prescription rates per 100,000, generalized estimating equations negative binomial regression models were used to account for repeated observations of states with a log link and log population offset. For male pre-exposure prophylaxis-to-need-ratio, we used generalized estimating equations linear models to account for repeated observations of states.
